# From subsidies to stressors: Positively skewed ecological gradients alter biological responses to nutrients in streams

**DOI:** 10.1002/eap.3086

**Published:** 2025-01-17

**Authors:** Stephen E. DeVilbiss, Jason M. Taylor, Matthew B. Hicks

**Affiliations:** ^1^ U.S. Geological Survey Lower Mississippi‐Gulf Water Science Center Oxford Mississippi USA; ^2^ United States Department of Agriculture – Agricultural Research Service, National Sedimentation Laboratory Water Quality & Ecology Research Unit Oxford Mississippi USA; ^3^ U.S. Geological Survey Lower Mississippi‐Gulf Water Science Center Jackson Mississippi USA

**Keywords:** agricultural streams, assemblage response, ecological criteria, eutrophication, Lower Mississippi River Basin, restoration

## Abstract

Subsidy–stress gradients offer a useful framework for understanding ecological responses to perturbation and may help inform ecological metrics in highly modified systems. Historic, region‐wide shifts from bottomland hardwood forest to row crop agriculture can cause positively skewed impact gradients in alluvial plain ecoregions, resulting in tolerant organisms that typically exhibit a subsidy response (increased abundance in response to environmental stressors) shifting to a stress response (declining abundance at higher concentrations). As a result, observed biological tolerance in modified ecosystems may differ from less modified regions, creating significant challenges for detecting biological responses to restoration efforts. Using the agriculturally dominated Mississippi Alluvial Plain (MAP) ecoregion in Mississippi, USA, as a case study, we tested the hypothesis that macroinvertebrate taxa that typically display a subsidy response to nutrient enrichment in less modified ecoregions (i.e., nutrient‐tolerance) shift to a stress response to increasing nutrients in highly modified watersheds with elevated baseline nutrient conditions (i.e., nutrient intolerance). The abundance and diversity of MAP‐specific intolerant taxa identified with threshold indicator taxa analysis were either unresponsive or exhibited a subsidy response to increasing nutrients in less modified ecoregions in Mississippi with less land alteration and lower nutrient concentrations, but declined at higher concentrations, providing evidence for a stress response to elevated nutrients in the MAP. Additionally, MAP‐specific tolerant and intolerant taxa richness responded to increased nutrients predictably and consistently across space and time within the MAP. However, in MAP streams, elevated specific conductance was predicted to dampen the response of tolerant and intolerant taxa richness to increasing nutrient concentrations, highlighting the importance of considering multistressor interactions when interpreting biological data. Lastly, we demonstrate the efficacy of this approach with sediment bacterial communities characterized with amplicon sequencing, which lack sufficient life history characteristics necessary for the development of multimetric indices. Both macroinvertebrate and bacterial communities responded similarly to increasing nutrient concentrations, suggesting DNA‐based approaches may provide an efficient biological assessment tool for monitoring water quality improvements in highly modified watersheds.

## INTRODUCTION

Human population growth is driving the conversion of natural landscapes to highly modified urban and agricultural ecosystems, which may have substantial and long‐lasting effects on aquatic habitats at global scales (Foley et al., 2005; Song et al., 2018). For example, nutrient enrichment from excess fertilizers and wastewater drives eutrophication, fuels harmful algal blooms, and leads to hypoxia and the formation of “dead zones,” with dissolved oxygen (DO) concentrations too low to sustain aquatic life (Daniel et al., 1998; Dodds, 2006; Lapointe et al., 2015). Many factors including internal nutrient loading, altered food web interactions, diffuse nutrient sources, and impacts of climate change can prevent the full recovery of aquatic and estuarian ecosystems, resulting in partial recovery or new degraded ecological conditions after restoration (Duarte et al., 2015; Jeppesen et al., 2005; Kemp et al., 2009; McCrackin et al., 2017; Nifong et al., 2022; Zhao et al., 2020). When perturbation occurs at the regional scale, the likelihood of full ecosystem recovery may be further hampered by reductions in regional species pools or loss of irreplaceable habitat features that support ecosystem structure and function, making biological assessment of recovery difficult (Isbell et al., 2013; Moreno‐Mateos et al., 2012; Taylor et al., 2023). Furthermore, widespread perturbation can lead to environmental gradients that are skewed toward higher stress at regional scales and may induce biological responses that differ from less modified regions (Taylor et al., 2023).

Odum et al.'s (1979) subsidy–stress gradient provides a useful framework for considering ecological responses to and recovery from perturbation. The subsidy–stress concept describes how ecosystem components can first be enhanced by low‐level increases in an environmental driver (subsidy) but become depressed at higher levels of the same variable (stress). Using nutrient loading and eutrophication as an example, certain species increase in abundance in response to elevated nutrient concentrations as productivity at the base of food webs is relieved from nutrient limitations (Elwood et al., 1981; King & Richardson, 2007; Tank & Dodds, 2003). However, substantially elevated baselines in highly modified ecological systems may push those same tolerant or unresponsive taxa past optimal conditions, resulting in declines in abundance from either direct or indirect responses to increasing nutrients, indicating a shift from a subsidy to stress response (King & Richardson, 2007; Odum et al., 1979; Taylor et al., 2023). Thus, biological tolerance can be context‐specific. The same species may appear either tolerant or intolerant to a particular environmental variable depending on system‐specific gradients. Considering how subsidy–stress responses may shift in highly modified regions requires different approaches for establishing taxa tolerance to monitor responses to best management practices designed to improve watershed health.

Considerable resources are allocated to restoring or improving the health of degraded aquatic ecosystems, but monitoring and assessment of potential improvements are challenging (BenDor et al., 2015; McCrackin et al., 2017). Benthic macroinvertebrate multimetric indices are a standard tool used to assess stream health and responses to restoration efforts and often incorporate measures of biological tolerance to environmental stressors (Bressler et al., 2006; Chutter, 1972; Hillsenhoff, 1987; Lenat, 1993). However, if biological tolerance derived from less modified systems is applied to highly modified systems with elevated ecological gradients, they may misrepresent the ecological realities of highly modified regions due to the absence of intolerant species and potential altered directionality of biological responses at higher ends of subsidy–stress gradients (Taylor et al., 2023). More sensitive metrics tailored to highly modified conditions are needed to detect small improvements in ecological health associated with restoration or best management practices.

**FIGURE 1:**
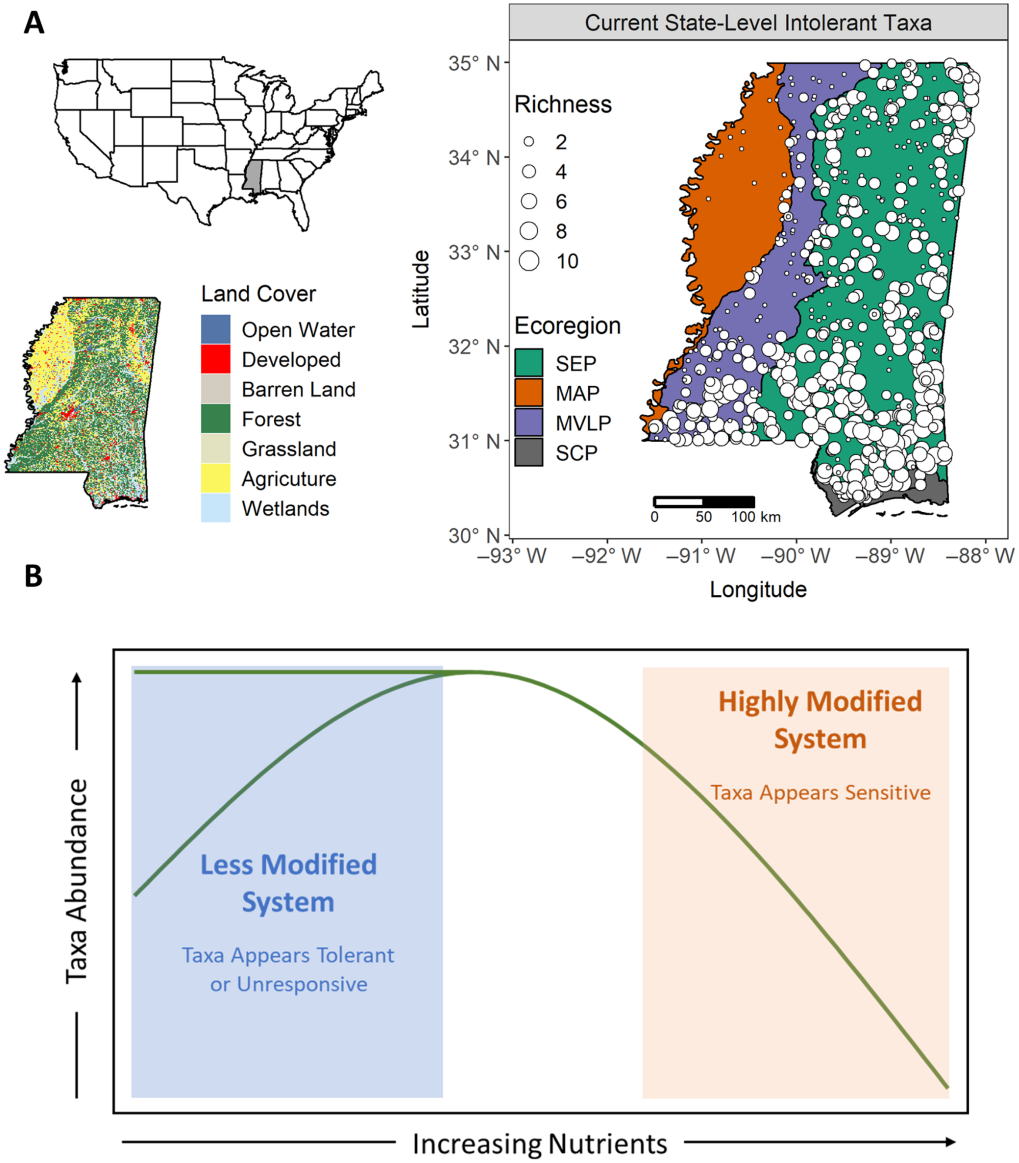
(A) Geographic location and land cover of Mississippi, USA, and the distribution and taxa richness of state‐level intolerant macroinvertebrate taxa (tolerance value ≤3, Bressler et al., 2006) used to develop and calibrate the Mississippi Benthic Index of Stream Quality (M‐BISQ) for monitoring and assessment purposes. MAP, Mississippi Alluvial Plain; MVLP, Mississippi Valley Alluvial Plain; SCP, Southern Coastal Plain; SEP, Southeastern Plain. (B) A theoretical hypothesis based on the subsidy–stress concept depicting how elevated nutrient baselines can cause a shift from a subsidy response (or no response, blue) to a stress response (orange).

In the state of Mississippi, USA, the Mississippi Alluvial Plain (MAP) ecoregion represents a highly modified agroecosystem with elevated nutrient gradients (Taylor et al., 2023) (Figure 1A). Compared with other ecoregions in Mississippi, current state‐level intolerant taxa (Bressler et al., 2006) used for biological assessment of streams are largely extirpated from the MAP (Figure 1A), which has prevented the development of reliable biomonitoring tools to detect improvements related to management efforts in the region. However, our recent work has demonstrated that macroinvertebrate taxa are indeed responsive to water quality gradients in the MAP, but responses occur at much higher concentrations than in the rest of the state and are driven by a unique subset of taxa (i.e., taxa that occur across the entire state but primarily only respond to the elevated stressors found in the MAP) (Taylor et al., 2023). Here, we address the need for biomonitoring tools in highly modified MAP streams by integrating previously identified MAP‐specific taxa that are either tolerant or intolerant to nitrogen (N) and phosphorus (P) into biological monitoring tools that detect subtle ecosystem improvements in highly modified systems that might exhibit nonlinear recovery trajectories.

We hypothesized that certain taxa that are tolerant or unresponsive to environmental stressors in other ecoregions of MS may be pushed from subsidy to stress responses in the MAP and represent currently unrecognized MAP‐specific intolerant taxa that could be useful for detecting subtle improvements in streams with elevated nutrient concentrations (Figure 1B). We also hypothesized that interactions with additional stressors (e.g., dissolved ions) will alter biological responses to nutrients and should be considered when developing stressor–response relationships. Lastly, we hypothesized that because bacterial communities have substantially higher diversity than macroinvertebrates and also respond to MAP water quality gradients (DeVilbiss et al., 2023), they may be more sensitive and effective at capturing subtle ecological changes to water quality improvements in highly modified streams.

## METHODS

### Study region

The MAP ecoregion is delineated by the historic floodplain of the Mississippi River. Before settlement in the late 1800s, the MAP was the largest continuous wetland in North America. In MS, the MAP was historically a dense, bottomland hardwood forest but was clear‐cut by early European settlers for the valuable cypress (*Taxodium distichum*) and tupelo gum (*Nyssa aquatica* and *Nyssa sylvatica*) timber. The MAP was subsequently drained and converted to row crop agriculture due to the highly productive Mississippi River alluvium deposits (Davis, 2016; Oswalt, 2013). The MAP is currently ~65% agricultural land, with much of the remaining land too low‐lying to drain and farm (Heintzman et al., 2024; Taylor et al., 2023; Figure 1A). In addition to considerable land cover alterations, an extensive levee system was constructed that hydrologically disconnected the MAP from the Mississippi River (Alexander et al., 2012). Nutrients and dissolved ions are elevated region‐wide in the MAP and show seasonal patterns consistent with agricultural practices (Shields & Knight, 2012; Taylor et al., 2023). There are no numerical water quality or habitat data for the region pre‐agriculture, which further complicates detecting and measuring recovery trajectories from management efforts. Given the region‐wide land cover and hydrologic alterations, ecological properties have likely shifted from pre‐agricultural reference conditions, making the MAP an ideal study region for developing new approaches for monitoring and assessing streams in highly modified regions where ecosystem recovery endpoints are uncertain.

### Calibration dataset

This work leverages two independent macroinvertebrate and water quality datasets. The first dataset, referred to as the calibration dataset, is a spatially extensive, long‐term dataset (*n* = 982 stream reaches throughout Mississippi) jointly collected by the U.S. Geological Survey (USGS) and Mississippi Department of Environmental Quality (MDEQ). Each stream reach in the calibration dataset was sampled once in a late summer sampling period for the MAP region (August–September, *n* = 99) and in winter for the rest of the state (December–February, *n* = 883) during a 12‐year period (2003–2015). The MAP is sampled during the late summer because that is the only time of year when streams are wadable. Although sampling periods between the MAP and the rest of the state differ, MAP macroinvertebrate assemblages are dominated by multivoltine taxa and our previous work demonstrates that ecoregional differences in macroinvertebrate assemblage structure are greater than seasonal variability within MAP streams (Taylor et al., 2023). Additionally, although the calibration dataset was collected over several years, the extent of agricultural land is stable in the MAP, but with some field‐scale variability associated with crop rotations and is dominated by soybean cultivation (Heintzman et al., 2024).

At each sampling reach, in‐stream chemical data (DO, pH, temperature, specific conductivity [SC], and turbidity) were collected using a multiprobe water quality meter and turbidimeter following MDEQ's surface water‐quality monitoring protocols (MDEQ, 2013). Surface water samples for total nitrogen (TN), total phosphorus (TP), total organic carbon (TOC), and alkalinity (Alk) were collected and acidified with H_2_SO_4_, stored on ice, and shipped to the MDEQ laboratory (Pearl, MS) for analyses within 24 h. Analysis of TN, TP, TOC, and Alk was performed in accordance with USEPA Methods for Chemical Analysis of Water and Wastes (USEPA, 1983) and *Standard Methods for Examination of Water and Wastewater* (Eaton et al., 2005) (TN method detection limit = 0.05 mg L^−1^, TP method detection limit = 0.02 mg L^−1^). Values below the detection limit were reported as half of the detection limit. Macroinvertebrate communities were sampled according to MDEQ's semiquantitative, rapid bioassessment protocols using a D‐frame net (800 × 900 μm mesh), following a low‐gradient/pool stream‐specific 20‐jab aquatic dip net protocol (MDEQ, 2013). To increase efficiency and reduce the costs of statewide biomonitoring programs, macroinvertebrate samples were sorted using a 200‐organism fixed‐count method. While studies suggest full counts may increase the probability of collecting rare species, multiple studies have demonstrated that the strength of macroinvertebrate assemblage–environmental relationships increased with increased fixed count sample size up to 200 individuals (Carter & Resh, 2001; King & Richardson, 2002; Ligeiro et al., 2020). Most individuals were identified to the genus level. Ten percent of all samples were identified by a second taxonomist to verify identification and ensure accuracy and consistency were maintained across samples (MDEQ, 2023). Prior to statistical analyses, we used a combination of Remove Parent or Merge Child (RPMC) depending on abundance and Distribute Parents Among Children (DPAC) approaches described by Cuffney et al. (2007) to resolve any taxonomic ambiguities. Ambiguous taxa were resolved based on ecoregional groupings. The RPMC and DPAC methods and resulting operational taxa units (OTUs) provide a balanced approach to preserving the most richness and abundance information while standardizing macroinvertebrate datasets for statistical analysis (Cuffney et al., 2007).

### Validation dataset

The second water quality and macroinvertebrate dataset, referred to as the validation dataset, was collected in MAP streams only (*n* = 27) in August–September of 2021 and is independent of the calibration dataset. Sites were chosen based on water quality data from the calibration dataset to represent the widest TN and TP gradients in the MAP. Water quality and macroinvertebrate sampling and processing methodologies were identical to the methods used to collect the calibration dataset. Macroinvertebrate samples were taxonomically classified by Rhithron Associates (Missoula, MT). Habitat data including wetted width, bankful width, bank height, average stream reach depth (averaged over 10 transects), particle size (measured with a digital hydrometer; see DeVilbiss et al., 2023 for method details), and densiometer readings were collected. Continuous dissolved oxygen (DO) sensors (miniDOT sensors, PME, Vista, CA, USA) were also deployed for ~1 month at select sampling sites where historical data from the calibration dataset indicated low, moderate, and high nutrient concentrations in order to better understand the relationship between nutrients, DO dynamics, and macroinvertebrate assemblages.

### Bacterial dataset

To test the potential use of our approach on alternative biological assemblages, we collected sediment samples at the same time and location as macroinvertebrate and water quality samples at all sites from the validation dataset in 2021 to characterize stream bacterial communities (DeVilbiss et al., 2023). Sediment samples were collected by inverting sterile, DNA‐free, 50‐mL Falcon™ tubes into the top 2 cm of sediment. We collected five subsamples per stream reach that were representative of the relative proportions of sand, silt, and clay. Subsamples were combined in sterile Whirl‐pak bags, homogenized, and stored on dry ice until returned to the U.S. Department of Agriculture—Agricultural Research Service National Sedimentation Laboratory (Oxford, MS) where they were stored at −80°C until processing. Raw bacterial sequencing data that support this research can be accessed at the NCBI SRA database (https://www.ncbi.nlm.nih.gov/sra) under accession number PRJNA909314.

### DNA extraction and sequencing

We extracted DNA from homogenized sediment samples using the Qiagen DNeasy PowerSoil Pro Kits (Qiagen, Inc, Valencia, CA, USA) following manufacturer protocols. Using polymerase chain reaction (PCR), we amplified the V4 region of the 16S rRNA gene in accordance with the Earth Microbiome Project protocol (Thompson et al., 2017, earthmicrobiome.org/protocols-and-standards/16s/). Each reaction well contained 23.5 μL Hot Start Mastermix (ThermoFisher Scientific), 0.5 μL of both the barcoded 515F (5′‐GGA CTA CNV GGG TWT CTA AT‐3′; Parada et al., 2016) and the 806R (5′‐GTG YCA GCM GCC GCC GTA A‐3′, Apprill et al., 2015) primers, and 1 μL of DNA template. Each sample was amplified in triplicate with a nontemplate control well to detect any contamination. For PCR, the thermocycler was initially held at 94°C for 3 min followed by 35 cycles of 94°C for 45 s, 50°C for 60 s, and 72°C for 90 s. Following 35 cycles, the thermocycler was held at 72°C for 10 min. We purified pooled triplicate PCR products using the Qiagen Qiaquick purification kit following manufacturer protocols. We quantified DNA concentrations in pooled triplicate PCR reactions using a Qubit fluorometer following manufacturer protocols. Lastly, we combined samples at equimolar concentrations and shipped the final pooled sample overnight on dry ice to the Duke Center for Genomic and Computational Biology (Durham, NC, USA). Samples were sequenced on an Illumina Miseq using 250 bp, paired‐end reads.

### DNA sequence quality filtering

We quality‐filtered DNA sequences in R using DADA2 (v1.22.0) (Callahan et al., 2016). Quality scores for both forward and reverse reads were high and were thus not trimmed or truncated prior to modeling error rates. We discarded individual reads with expected errors higher than 2 (MaxEE = 2) and truncated the remaining reads at the first base with a quality score ≤2 (truncQ = 2) using the *filterAndtrim* function. Error rates were learned on quality‐filtered reads using the *learnErrors* function and identified errors were subsequently removed using the *dada* function. We used the *removeBimeraDenovo* function to merge paired reads and remove chimeras. Amplicon sequence variants (ASVs) were taxonomically classified using the *assignTaxonomy* function, which uses a naïve Bayesian classifier (Wang et al., 2007) and the Silva training set (v138.1) (Quast et al., 2013). We used an internal maximum likelihood method to construct a phylogenetic tree with the phangorn (v2.9.0) (Schliep, 2011) and DECIPHER (v2.22.0) (Wright, 2016) packages. Lastly, we removed ASVs that were only observed once in the dataset or occurred in only one sample, as well as ASVs taxonomically classified as chloroplast, mitochondria, or unassigned at the Domain level. The final ASV table consisted of 7092 individual ASVs.

### Threshold indicator taxa analysis

Threshold indicator taxa analysis (TITAN, Baker & King, 2010) combines Indicator Species Analysis (IndVal; Dufrene & Legendre, 1997) and nonparametric changepoint analysis to (1) identify taxa that are either tolerant or intolerant to specific environmental gradients, (2) quantify the regions of environmental gradients where the abundance of individual taxa change, and (3) sum individual taxa responses to identify the region of environmental gradients that induces the largest assemblage‐level change. For this study, we ran TITAN on the bacterial dataset only to identify bacterial nutrient indicators. We performed TITAN on bacterial ASV sequence read counts, removing all ASVs with fewer than three observations prior to analyses in accordance with TITAN protocol. We used 500 permutations and 500 bootstrapped replicates. Only bacterial ASVs that responded in the same direction across gradients (i.e., purity) and were significantly different from a random distribution (at *p* < 0.05; i.e., reliability) in 95% of bootstrapped replicates were considered to be robust tolerant or intolerant ASVs that were retained for further analyses.

We previously used TITAN on abundance data from the calibration dataset (see Taylor et al., 2023) to compare ecoregional differences in assemblage‐level responses with water quality gradients in Mississippi. Here, we expand on that work by calculating metrics (indicator taxa richness and relative abundance) from TITAN‐derived, MAP‐specific tolerant and intolerant macroinvertebrate taxa from the calibration dataset (Taylor et al., 2023). MAP‐specific nutrient indicator taxa identified by TITAN in the calibration dataset were extracted from the validation dataset (i.e., TITAN was not run on the validation dataset) to compare responses across nutrient gradients between datasets and test whether results from a larger, long‐term dataset were applicable to a smaller dataset typical of a state monitoring dataset. Additionally, we developed the same metrics based on TITAN‐derived tolerant and intolerant bacterial ASVs in order to compare bacterial metric responses to those based on macroinvertebrates.

### Testing the subsidy–stress hypothesis

We calculated the richness (i.e., total number of unique taxa) and relative abundance of MAP‐specific TN and TP‐intolerant macroinvertebrate taxa for all 982 observations across Mississippi. We used a 95th percentile polynomial regression (quantreg R package, Koenker, 2023) to test the relationships between MAP‐specific intolerant taxa and statewide TN and TP gradients and visualize how increasing nutrients affects the upper limit of MAP‐specific intolerant‐taxa relative abundance and richness in all ecoregions of Mississippi. Data were fit with 95th percentile regressions because stressors like nutrients often limit the maximum abundance or diversity of an organism/assemblage rather than the mean or minimum.

### Generalized additive modeling

We used generalized additive models (GAMs) to describe changes in TITAN‐derived taxa richness along TN and TP gradients. Our goal in characterizing the richness of TITAN‐derived taxa along nutrient gradients was not to identify threshold responses, but rather to identify subtle, gradual improvements in the diversity of nutrient‐responsive taxa that may occur at concentrations than assemblage thresholds and provide an early indication that nutrient reduction efforts are having an impact. All analyses were performed in R using the mgcv and gratia packages (Simpson, 2023; Wood, 2011). Prior to modeling, tolerant and intolerant taxa and ASV richness (i.e., total number of unique taxa or ASVs identified by TITAN) were calculated for use as the response variable. We fit GAMs using the *gam* function with a negative binomial distribution (log link) using the restricted maximum likelihood (REML) method. Comparison with other distributions that allow for overdispersion (Poisson, quasi‐Poisson) using Pearson's dispersion coefficient indicated that a negative binomial distribution reduced overfitting and provided the best model fits. Additionally, total tolerant/intolerant abundance and percent tolerant/intolerant abundance were calculated and tested as response variables, but richness consistently produced the best model fits and was chosen as the response variable for further analyses. We used the *derivatives* function (gratia package in R) to calculate the first derivative of each GAM. Conceptually, the derivative indicates how much taxa or ASV richness is changing at each concentration across observed TN and TP gradients and any region of the gradient where the derivative CI overlaps zero indicates a zone of no change (e.g., Simpson, 2018; Sonderegger et al., 2008).

### Testing multistressor interactions

We explored potential interactions among nutrients and SC, another stressor of concern in the MAP driven largely by extensive irrigation with high‐conductivity ground water (Kresse & Clark, 2008). The effects of TN or TP, SC, and interactions among TN or TP and SC and tolerant and intolerant macroinvertebrate richness were explored using tensor interactions in GAMs (e.g., richness ~ s(TN) + s(SC) + ti(TN, SC) and richness ~ s(TP) + s(SC) + ti(TP, SC)), using the calibration dataset. Prior to analysis, nutrient and SC values were log‐transformed, scaled, and centered due to differences in variable ranges that differ by orders of magnitude. Detailed habitat data were not collected in the calibration dataset, and there were not enough samples in the validation dataset for sufficient statistical power to analyze interactions with habitat variables. However, correlations among water quality, habitat, and macroinvertebrate metrics were analyzed in the validation dataset to assess the potential influence of habitat characteristics on macroinvertebrate responses to nutrients.

## RESULTS

### Macroinvertebrate TITAN

Macroinvertebrate taxa identified by TITAN as either tolerant or intolerant to TN and TP gradients in the MAP can be found in Table 1 and Appendix S2: Figure S1. TITAN summary statistics including the number of responsive taxa, maximum sum(*z*) values, thresholds, and CIs are listed in Table 2. Numerous taxa identified by TITAN as either tolerant or intolerant to TN and TP in the MAP were not identified as responsive to nutrients by TITAN in other ecoregions of MS, despite inhabiting streams across the state (Table 1; Appendix S2: Figure S1) (see Taylor et al., 2023 for TITAN for other MS ecoregions). Numerous TITAN‐derived tolerance designations for the MAP also do not match tolerance values developed for macroinvertebrates in non‐MAP regions of MS (Table 1; Bressler et al., 2006). Specifically, six TN‐tolerant and eight TN‐intolerant taxa were only responsive to MAP TN gradients. Additional two taxa, *Baetis* and *Tricorythodes*, responded negatively to TN in the MAP but positively in other ecoregions. For TP, 11 intolerant and seven tolerant taxa were only responsive to MAP TP gradients, while two additional taxa, *Baetis* and *Branchiura*, responded negatively in the MAP but positively in other ecoregions (Table 1; Appendix S2: Figure S1).

**TABLE 1:** Taxonomy of macroinvertebrate taxa identified by threshold indicator taxa analysis (TITAN) as tolerant and intolerant to total nitrogen (TN), total phosphorus (TP), or both nutrients.

Taxa	TN	TP	MS tolerance value
Tolerant			
Acariformes*	X		…
Bratislavia*	X		…
Clinotanypus*	**X**		**4.5**
**Dubiraphia**		**X**	**4.5**
Epitheca*		X	…
Erpobdellidae*		X	…
Glossiphoniidae	X	X	…
Glyptotendipes	X		9
Goeldichironomus*	X	X	…
Gyraulus*		X	…
**Hydrobiidae**	**X**	**X**	**3.9**
Ischnura	X		9.7
Libellulidae	X	X	7.2
Palaemonidae*	**X**		**2**
Peltodytes	X	X	8.2
**Physidae**	**X**	**X**	**6.5**
Pisidiidae	X	X	…
Planorbella		X	…
**Stenelmis**		**X**	**4.8**
Tanypus*	X		…
Tropisternus*		**X**	**6.4**
Zavreliella*		X	…
Intolerant			
Acerpenna*	**X**	**X**	**5.9**
Atractides*		X	…
Baetis*	**X**	**X**	**3.6**
**Branchiura**		**X**	**10**
**Calopteryx**		**X**	**5.6**
Centroptilum/Procloeon*		**X**	**7.7**
Chimarra	X		1.2
Cladotanytarsus*	**X**	**X**	**3.8**
Corydalus*	**X**		**3.7**
**Corynoneura**	**X**	**X**	**3.2**
Cricotopus*	**X**	**X**	**5.7**
Gomphus*		**X**	**5.2**
**Helichus**		**X**	**5.0**
Hydropsyche		X	3.0
Hygrobates*	X	X	…
Labiobaetis*	X	X	…
Lopescladius		X	…
**Maccaffertium**	X	X	…
Macromia*	**X**	**X**	**4.9**
Nectopsyche	**X**	**X**	**5.5**
Paracloeodes*	X	X	…
**Progomphus**	**X**	**X**	**6.5**
**Rheotanytarsus**	**X**	**X**	**3.3**
Saetheria*	X	X	…
**Simuliidae**	**X**	**X**	**3.5**
Stempellinella	X	X	1.6
**Stenonema**	**X**	**X**	**4.2**
**Tanytarsus**	**X**		**3.5**
**Thienemanniella**	**X**	**X**	**4**
**Thienemannimyia**	**X**	**X**	**5.8**
Tricorythodes	X	X	2.2
Zavrelimyia*	**X**	**X**	**5.6**

*Note*: Taxa in boldface do not match tolerance designations developed based on data collected in other Mississippi ecoregions (Mississippi Tolerance Value from Bressler et al., 2006). An asterisk indicates taxa that were not responsive to TN or TP in other Mississippi ecoregions based on TITAN (see Taylor et al., 2023).

Abbreviations: TN, total nitrogen; TP, total phosphorus.

**TABLE 2:** Comparison of calibration and bacterial dataset threshold indicator taxa analysis (TITAN) max sum(*z*), changepoints, 5–95 quantiles, and number of macroinvertebrate taxa/bacterial amplicon sequence variants (ASVs), respectively, from which thresholds were derived.

Dataset	TN		TP	
	Sum(z–)	Sum(z+)	Sum(z–)	Sum(z+)
Calibration inverts (*n* = 99)	132.6; 0.42; 0.28–0.62; 24	52.4; 0.88; 0.78–1.07; 14	147.8; 0.11; 0.07–0.13; 29	54.0; 0.16; 0.15–0.30; 15
Bacteria (*n* = 28)	135.6; 0.50; 0.41–0.55; 28	219.0; 0.50; 0.41–0.58; 70	178.0; 0.09; 0.08–0.15; 39	177.3; 0.08; 0.08–0.13; 46

*Note*: The TITAN analysis of the calibration dataset is from Taylor et al. (2023) but is summarized here to compare bacterial and macroinvertebrate responses. *z*−, intolerant; *z*+, tolerant. Max sum(*z*) values indicate the TP or TP concentration where the largest number of individual taxa within each assemblage responded.

Abbreviations: TN, total nitrogen; TP, total phosphorus.

### Subsidy–stress response of MAP tolerance indicator taxa to state‐wide nutrient gradients

**FIGURE 2:**
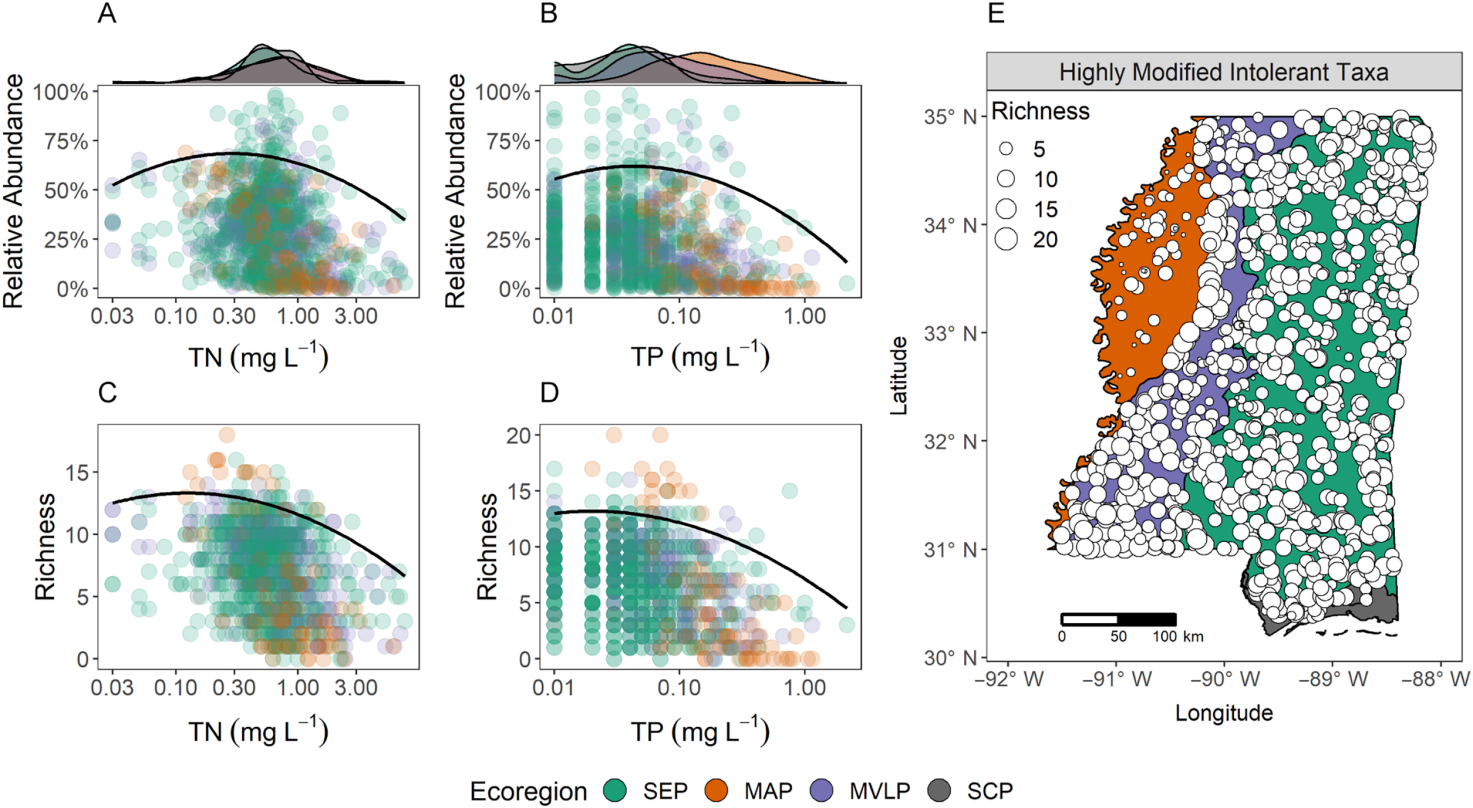
Responses of taxa relative abundance and richness for macroinvertebrate taxa from the calibration dataset identified by threshold indicator taxa analysis (TITAN) as intolerant to (A, C) total nitrogen (TN) and (B, D) total phosphorus (TP) in the Mississippi Alluvial Plain (MAP). Curves represent 95th quantile polynomial regressions and density plots on the upper axis show the distribution of nutrient concentrations among ecoregions. (E) The state‐wide distribution and richness of macroinvertebrate taxa that were identified as intolerant to either TN or TP in the MAP.

The relative abundance and richness of taxa identified as intolerant to TN and TP in the MAP responded to statewide nutrient gradients that included less modified regions, in a manner consistent with our subsidy–stress hypothesis (Figure 2). For both TN and TP, the relative abundance of intolerant taxa displayed a subsidy–stress relationship, with relative abundance increasing at lower concentrations but decreasing as concentrations reached the upper end of the gradient (Figure 2A,B). Taxa richness of TN and TP‐intolerant taxa did not change with increasing concentration at the lower end of the gradient, but began to decline toward the upper end of the gradient (Figure 2C,D). It is important to note that the resolution of TP concentrations at the lower end of the gradient is limited by the detection limit of 0.02 mg L^−1^, and thus the true response of MAP‐specific TP‐intolerant taxa may differ from our observations if detection limits were decreased. Additionally, while the relative abundance of MAP‐intolerant taxa was the greatest in the Southeastern Plains (SEP) ecoregion, taxa richness was greatest in the MAP. Compared with current state‐level intolerant taxa (Figure 1A), the diversity of MAP‐specific intolerant taxa was much higher across the entire state of MS, including the MAP (Figure 2E).

### Macroinvertebrate responses across MAP‐specific nutrient gradients

**FIGURE 3:**
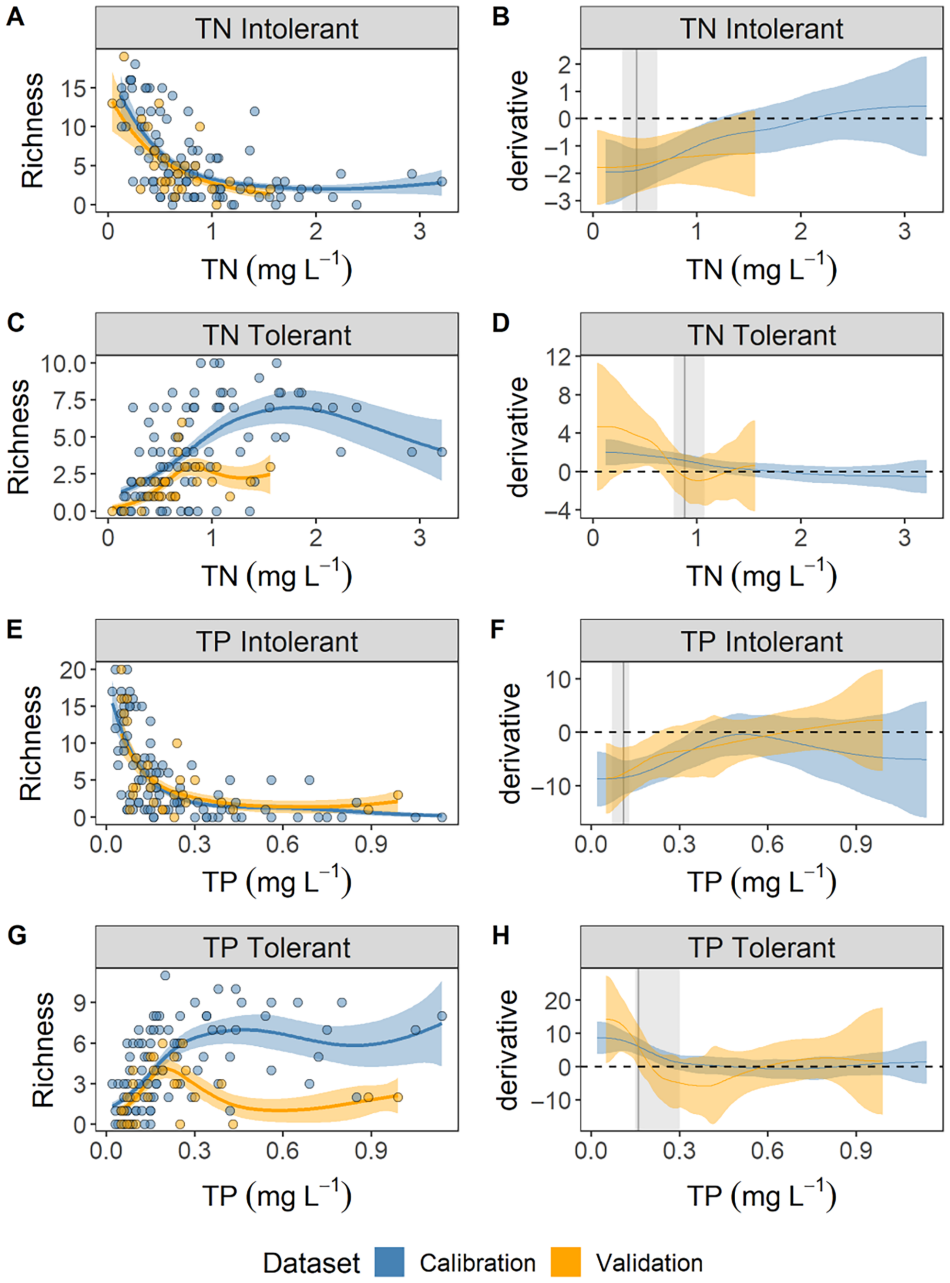
Generalized additive model (GAM) (A, C, E, G) fits and (B, D, F, H) first derivatives for taxa richness of intolerant and tolerant taxa identified by threshold indicator taxa analysis (TITAN) for the Mississippi Alluvial Plain (MAP) across total nitrogen (TN, A–D) and total phosphorus (TP, E–H) gradients. Orange points and 95% CIs are from the calibration dataset (*n* = 99) that was used to identify tolerant and intolerant taxa with TITAN. Blue points and 95% CIs are from a separate, independent dataset collected in the summer of 2021 (*n* = 27) that was used to validate analyses and response curves from the Mississippi Benthic Index of Stream Quality (M‐BISQ) dataset. Any point across the gradient where derivative CIs do not overlap 0 indicates a zone of change in taxa richness. The grey vertical line and shaded region in panels B, D, F, and H indicate TITAN thresholds and 95% CIs, respectively.

Tolerant and intolerant macroinvertebrate richness across TN and TP gradients was consistent between calibration and validation datasets. Although TP detection limits may have affected observed responses to TP at lower ends of the gradient observed in the SEP and MVLP, all TP concentrations in MAP streams, except one, were >0.02 mg L^−1^, suggesting detection limits did not influence our observed responses to nutrient gradients. Generally, intolerant taxa richness decreased, and tolerant richness increased with increasing nutrient concentrations (Figure 3A,C,E,G). Although tolerant taxa richness was more variable at higher TN and TP gradients, there was a consistent zone of rapidly decreasing richness as TN and TP concentrations increased (Figure 3C,G). GAM fits explained between 28.8% and 48.3% deviance (Table 3). First‐derivative analysis of each GAM delineated a zone of decreasing intolerant richness as TN increased up to 1.13 mg L^−1^ and increasing tolerant richness up to 1.07 mg L^−1^ TN for the calibration dataset (Table 3, Figure 3B,D). Concentrations where changes in richness occurred were similar for validation datasets, differing from the calibration dataset only by 0.14 mg L^−1^ for TN‐intolerant and 0.46 mg L^−1^ for TN‐tolerant (Table 3). Similarly, first‐derivative analysis of GAM responses to increasing TP identified a zone of decreasing intolerant richness up to 0.33 mg L^−1^ and a zone of increasing tolerant richness up to 0.25 mg L^−1^ (Table 3, Figure 3F,H). The initiation points of those zones for the validation dataset differed from the calibration dataset by 0.11 mg L^−1^ for TP‐intolerant, and 0.10 mg L^−1^ for TP‐tolerant (Table 3).

**TABLE 3:** Summary statistics of generalized additive models for the calibration, validation, and bacterial datasets.

Variable	Parametric coefficient		Smooth term		*R* ^2^	Deviance explained (%)	Point of change (mg L^−1^)
	*z*	*p*	χ^2^	*p*			
Calibration macroinvertebrates							
TN intolerant	20.62	<0.0001	56.86	<0.0001	0.46	39.7	<1.13
TN tolerant	1.26	<0.0001	41.01	<0.0001	0.34	28.8	>1.07
TP intolerant	1.39	<0.0001	77.21	<0.0001	0.50	47.0	<0.33
TP tolerant	1.31	<0.0001	52.15	<0.0001	0.38	35.3	>0.25
Validation macroinvertebrates							
TN intolerant	1.53	<0.0001	12.78	<0.001	0.44	40.8	<1.27
TN tolerant	0.55	<0.001	8.46	0.05	0.36	48.3	>0.61
TP intolerant	1.55	<0.0001	17.32	<0.001	0.47	45.7	<0.22
TP tolerant	0.84	<0.0001	13.88	0.01	0.36	37.4	>0.15
Bacteria							
TN intolerant	2.18	<0.001	18.57	<0.001	0.45	46.5	<0.70
TN tolerant	3.56	<0.0001	33.39	<0.0001	0.66	47.5	>0.61
TP intolerant	2.31	<0.0001	48.10	<0.0001	0.67	67.6	<0.25
TP tolerant	3.29	<0.0001	43.07	<0.0001	0.64	55.1	>0.17

*Note*: Point of change indicates the concentration at which the derivative 95% CI no longer overlaps zero.

Abbreviations: TN, total nitrogen; TP, total phosphorus.

In addition to nutrients, the richness of TN‐ and TP‐tolerant and intolerant taxa was also correlated with sediment particle size, pH, DO, SC, TOC, and Alk in the validation dataset (Appendix S2: Figure S2). In MAP streams, benthic substrates consist entirely of variable proportions of sands, silts, and clays, with median particle sizes throughout sampled stream reaches ranging from 1.08 μm (clay) to 429.98 μm (sand). Specifically, intolerant taxa richness increased with increasing particle size (toward mostly sand sediments), while tolerant taxa richness increased with decreasing particle size (toward mostly clay and silts). Intolerant richness was negatively correlated with SC, TOC, and Alk. Only TN‐tolerant richness was positively correlated with TOC and only TP‐tolerant richness was positively correlated with Alk. Intolerant richness for TP‐responsive taxa was negatively correlated with pH while TP‐tolerant richness was positively correlated. Instantaneous DO concentrations did not have strong correlations with TN‐ or TP‐intolerant or tolerant taxa, but measurements recorded at the time of sampling likely do not adequately characterize DO conditions to which macroinvertebrates are routinely exposed. Both the range of continuous DO concentrations and the minimum DO concentrations over 24 h vary predictably with nutrient concentrations, with lower nutrient streams exhibiting less DO variability and higher minimum concentrations and higher nutrient streams exhibiting much wider DO ranges and lower minimums, frequently falling below the MS state DO criteria of 4 mg L^−1^ for instantaneous measurements or 5 mg L^−1^ for daily averages (Appendix S2: Figure S3).

**FIGURE 4:**
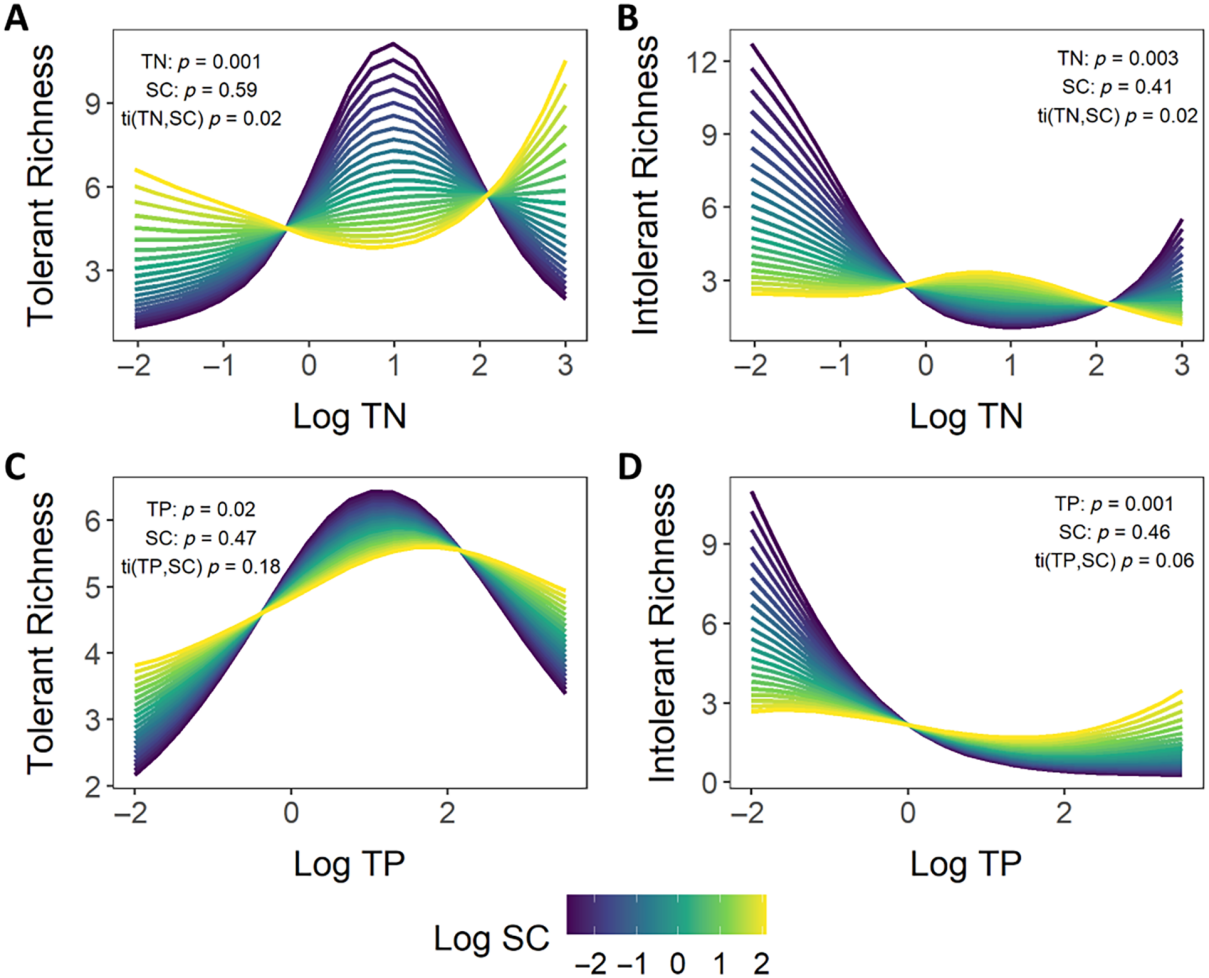
Interactive effects of specific conductivity (SC) and total nitrogen (TN) on (A) TN‐tolerant and (B) TN‐intolerant macroinvertebrate richness and the interactive effects of SC and total phosphorus (TP) on (C) TP‐tolerant and (D) TP‐intolerant macroinvertebrate richness.

We also explored interactions and the main effects of nutrients and SC on macroinvertebrate‐tolerant and ‐intolerant taxa richness using the full calibration dataset. While SC alone did not have a significant effect on either nutrient‐tolerant or ‐intolerant taxa richness, significant interactions between TN and SC were observed for both tolerant and intolerant taxa richness (both *p* = 0.02, deviance explained = 38.4 and 41%, respectively) (Figure 4A,B). For TP, a marginally significant interaction with SC was observed for intolerant taxa richness (*p* = 0.06, deviance explained = 24%), and no significant interaction with SC was observed for tolerant taxa richness (*p* = 0.18, deviance explained = 41.8%) (Figure 4C,D). For all significant interactions, the predicted response of tolerant and intolerant taxa richness to nutrients decreased with increasing SC. More specifically, predicted intolerant taxa richness decreased with increasing SC at the lowest nutrient concentrations, resulting in a dampened expected decline in richness across the gradient (Figure 4B,D). Conversely, for TN‐tolerant taxa, predicted richness consistently increased with increasing SC at low TN concentrations, resulting in a lesser expected increase in richness across the gradient (Figure 4A).

### Comparison of macroinvertebrate and bacterial responses

**FIGURE 5:**
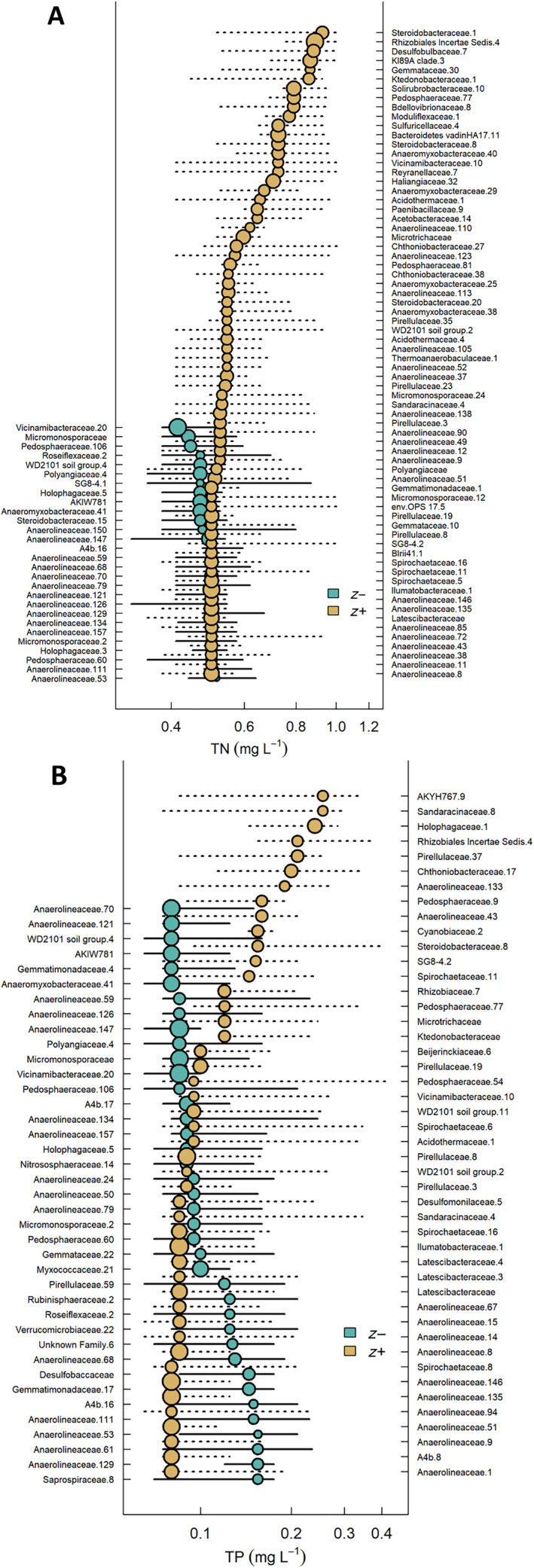
Pure and reliable sediment bacterial amplicon sequence variants (ASVs) taxonomically classified to the family level, which were identified by threshold indicator taxa analysis (TITAN) as intolerant (*z*−, blue circles) or tolerant (*z*+, orange circles) to (A) total nitrogen (TN) (B) or total phosphorus (TP).

**FIGURE 6:**
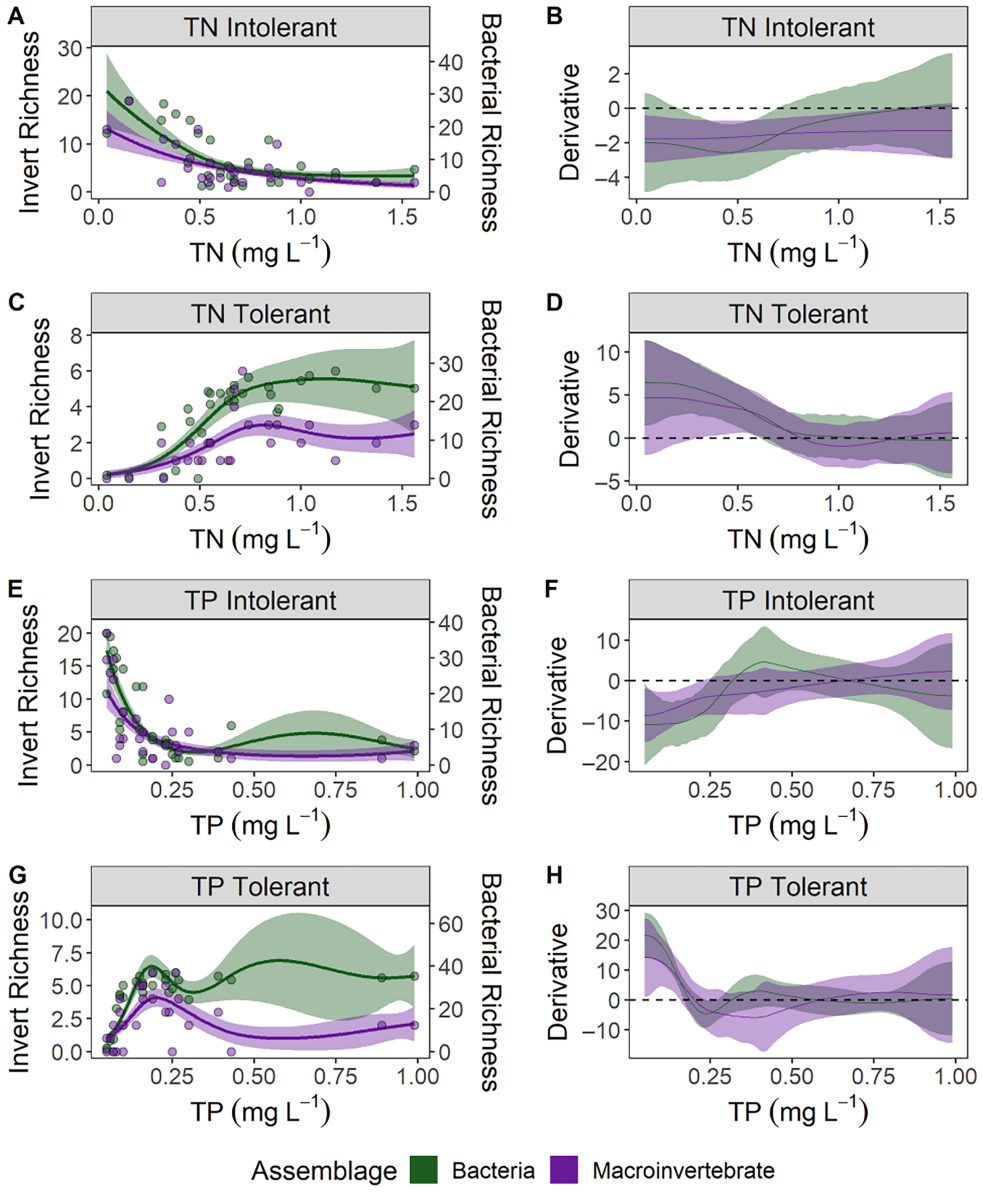
Generalized additive model (GAM) (A, C, E, G) fits and (B, D, F, H) first derivatives for taxa richness of intolerant and tolerant taxa identified by threshold indicator taxa analysis (TITAN) for the Mississippi Alluvial Plain (MAP) across total nitrogen (TN, A–D) and total phosphorus (TP, E–H) gradients. Purple points and 95% CIs are macroinvertebrate data from the dataset collected in the summer of 2021 (*n* = 27). Green points and 95% CIs are bacterial data collected at the same time and location as the macroinvertebrate data in the validation dataset. Any point across the gradient where derivative CIs do not overlap 0 indicates a zone of change in taxa richness. Note that macroinvertebrate and bacterial richness are on different scales.

For bacterial communities, TITAN identified 29 intolerant ASVs and 70 tolerant ASVs in response to increasing TN concentrations (Figure 5A). For TP, TITAN identified 39 intolerant ASVs and 46 tolerant ASVs (Figure 5B). Metrics based on TITAN‐intolerant and ‐tolerant ASVs responded similarly across TN and TP gradients to macroinvertebrate‐derived metrics collected at the same time and locations (2021 macroinvertebrate validation dataset; Figure 6). Based on GAMs and GAM derivatives of TITAN‐derived taxa richness, intolerant ASV richness begin to increase as TN concentrations are reduced to 0.70 mg L^−1^ or less and as TP concentrations are reduced to 0.25 mg L^−1^ or less (Table 2), which are substantially higher concentrations than TITAN thresholds that describe where maximum change is occurring based on summing individual ASV responses (0.50 mg L^−1^ for TN and 0.09 mg L^−1^ for TP). Except for TN‐intolerant AVS, improvements in both bacterial and macroinvertebrate diversity due to nutrient reductions are expected to begin occurring at similar nutrient concentrations (Table 3). Both TN‐tolerant macroinvertebrates and bacterial ASV diversity increased significantly up to 0.61 mg L^−1^ and TP‐tolerant taxa increased up to concentrations of 0.15 and 0.17 mg L^−1^ for macroinvertebrates and bacteria, respectively (Table 3, Figure 6C,D,G,H). The diversity of TP‐intolerant macroinvertebrates also responded across a similar concentration range up to 0.22 and 0.25 mg L^−1^ for macroinvertebrates and bacteria, respectively (Table 3, Figure 6E,F).

## DISCUSSION

### Shifted biological tolerance in highly modified watersheds

Biological organisms have optimal conditions or requirements for environmental variables that impact their tolerance to perturbation (e.g., DeVilbiss et al., 2022; Hokanson et al., 1973). Optimal ecological conditions have been observed with macroinvertebrate taxa across numerous environmental gradients, including P (King & Richardson, 2007), inorganic N (Wagenhoff et al., 2011), as well as sedimentation and land use (Niyogi et al., 2007; Wagenhoff et al., 2012). The subsidy–stress response (Odum et al., 1979) provides a theoretical explanation for shifted biological tolerance in highly modified watersheds due to elevated environmental gradients. Increasing nutrients above baseline conditions enhances food resources and alters consumer–resource relationships by favoring small‐bodied, r‐selected species with higher growth rates (i.e., tolerant species), which can reduce the competitive advantage of slower growing species with more specific habitat requirements (Cook et al., 2018; Cross et al., 2005; Evans‐White et al., 2009). Excess enrichment can increase the magnitude and duration of low DO and increase diel DO and associated pH swings, which may alter the toxicity of ionizable contaminants, thereby increasing physiological stress for all but the most tolerant taxa (Miltner & Rankin, 1998; Valenti et al., 2011). Consistent with this nutrient subsidy–stress concept, we determined that MAP‐specific intolerant taxa are found in streams across the state of Mississippi, but their relative abundance and diversity only begin to decline at elevated TN and TP concentrations observed most commonly in the MAP. Thus, in less modified regions like the SEP or MVLP, low‐level nutrient enrichment potentially subsidizes some MAP‐intolerant taxa, resulting in metrics (e.g., richness) that are either unresponsive or respond positively to increasing nutrients. Conversely, in highly enriched conditions characteristic of the modified streams in the MAP, correlated stressors (e.g., habitat limitation) and increased productivity potentially lead to physiological stress from DO swings (Appendix S2: Figure S3) that culminate in an intolerant or stress response of some of the same taxa, resulting in metrics that respond negatively to increasing nutrients.

Although subsidy responses at lower nutrient concentrations were not as pronounced as stress responses at high concentrations, the detection of biological responses at low concentrations in less‐altered ecoregions was hampered by relatively high method detection limits typical of state monitoring programs that resulted in many sites below detection. Thus, our analysis did not capture the response to the full state‐wide enrichment gradient (Taylor et al., 2014). While this somewhat inhibits our ability to completely confirm our first hypothesis, it does not impact our ability to utilize observed patterns in macroinvertebrate response to detect changes in nutrient impacts to MAP streams. In highly modified regions like the MAP, method detection limits may be less of a concern because almost all streams in the MAP have TP concentrations above the method detection limit, resulting in a more accurate characterization of taxa responses across representative nutrient gradients.

### Considerations for multistressor interactions

The effects of other environmental stressors should also be considered when interpreting biological responses to nutrients. We hypothesized that, due to the strong effect dissolved ions have on macroinvertebrate communities (Simonin et al., 2021; Timpano et al., 2018; Vander Vorste et al., 2019) and the higher overall SC in the MAP from groundwater pumping for irrigation (Kresse & Clark, 2008), SC likely interacts with nutrients altering macroinvertebrate community responses, and such interactions could have critical implications for the accurate interpretation of biological data. Our simple models predict that increasing SC dampens the response of macroinvertebrate taxa to increasing nutrients, similar to interactions observed in other regions (Kefford et al., 2022). Thus, signs of nutrient reduction may be more difficult to detect using macroinvertebrate community data in streams with elevated SC. Conversely, management efforts that target reductions in both nutrients and dissolved ions (e.g., tailwater recovery systems that reduce the pumping of saline groundwater for irrigation) may result in more substantial and easily detectable ecological improvements than efforts that only target nutrients. Therefore, future studies and biomonitoring in the MAP or other highly modified regions should also consider how other underlying environmental conditions (e.g., habitat, temperature) can impact observed responses to specific stressors of concern.

### Developing biomonitoring tools for highly modified streams

TITAN was developed to overcome challenges associated with identifying robust ecological community thresholds to novel environmental gradients (Baker & King, 2010). Traditional approaches like multimetric indices or indices of biotic integrity simplify community data by calculating aggregate metrics (e.g., % tolerant or intolerant taxa). TITAN individual responses of both tolerant and intolerant taxa and is thus more sensitive and provides more information than traditional approaches, which is critical for detecting more subtle responses of rare species contributions to assemblage change (Baker & King, 2010; Smucker et al., 2022), or as is the case in the MAP, responses of common species to truncated stressor gradients in highly modified watersheds with reduced regional taxa pools (Taylor et al., 2023). Some of the earliest applications of TITAN successfully identified biological assemblage responses at nutrient concentrations as low as 0.02 mg L^−1^ TP (LeBrun et al., 2018; Taylor et al., 2014). Conversely, we previously used TITAN to identify macroinvertebrate responses in eutrophic agricultural streams, which occurred at substantially higher TP concentrations of 0.11 mg L^−1^ (Taylor et al., 2023). These TITAN results provide biological support for regional nutrient reduction goals in the MAP but do not directly provide a means of monitoring biological improvements from restoration efforts in individual streams over time. However, TITAN identifies regionally‐specific taxa that are either intolerant or tolerant to water quality stressors (King & Baker, 2010). In the current study, we used these regionally‐specific nutrient‐tolerant and ‐intolerant macroinvertebrate taxa to develop simple monitoring metrics for evaluating change or nutrient condition of individual streams.

In highly modified watersheds, there is a need for biological assessment tools that are more sensitive to modest changes in biology than traditional multimetric indices while not relying on reference conditions for calibration, as reference conditions may not exist, and recovery trajectories may not be linear. In the MAP, we identified between 15 and 29 nutrient‐responsive taxa in the calibration dataset, many of which were uniquely responsive to MAP TN and TP gradients. We assumed that if responses of these taxa across TN and TP gradients are consistent throughout the MAP over time, our approach could offer a method for establishing a region‐specific list of taxa that can be used to assess the biological health of streams over time. To test this assumption, we compared the richness of taxa identified by TITAN as intolerant or tolerant to nutrient enrichment between a calibration and independent validation dataset to confirm metrics based on TITAN output respond consistently to nutrient gradients across space and time. Thus, from an applied standpoint, our approach could be used to establish regional management thresholds or biological classifications (e.g., best attainable, less impaired, impaired, most impaired) based on simple diversity–stressor relationships that individual streams could be assessed against (Stoddard et al., 2006).

In the absence of management classifications that are typical of highly modified regions like the MAP, a potential application of our results would be for water quality managers to track numbers of tolerant/intolerant taxa over time using trend analyses as evidence of biological responses to management efforts. For example, dedicated long‐term monitoring at stream sites downstream of agricultural areas experiencing different levels of adoption of best management practices could be used to estimate ecological responses to targeted management in the MAP. In the context of biomonitoring, it is important to consider the difference between threshold responses and signs of gradual change across gradients. A commonly observed artifact of aggregating individual taxa responses in a single metric like richness is the appearance of a wedge‐shaped distribution that can artificially drive perceived community responses toward higher concentrations (King & Baker, 2010). This smoothing effect caused by aggregating nonlinear responses could result in developing water quality goals that are not stringent enough to protect sensitive species. However, our goal of using a metric like richness was not to identify threshold responses at low concentrations, but rather to develop sensitive tools that can identify zones of gradual biological change at higher concentrations that indicate whether or not management efforts are driving the needle in the right direction, even if water quality goals have yet to be attained. The zones of biological change across nutrient gradients in the calibration and validation dataset were largely consistent, providing further support for the robustness of our approach. Additionally, the region of GAM derivative curves most different from zero for intolerant taxa (indicating zone of maximum decline) corresponded well with TITAN sum *z‐*assemblage thresholds, suggesting the sensitivity of our aggregate metric benefited from identifying and characterizing the responses of a suite of sensitive taxa specific to the current conditions in the MAP (King & Baker, 2010).

The biggest discrepancies in biological responses to nutrients between the calibration and validation dataset were with tolerant taxa at higher concentrations. This may be the result of the validation dataset not representing the high end of the gradient uniformly (Appendix S2: Figure S4) or multiple stressor interactions like SC at high concentrations, which have been documented (Beermann et al., 2018; Davis et al., 2018; Kefford et al., 2022). Regardless, responses at lower concentrations and thus zones of biological change were largely consistent between datasets, providing support for our approach to developing tools for monitoring the response of streams to nutrient reduction efforts in highly modified ecoregions. This is relevant because significant time lags can occur between the implementation of best management practices and measurable nutrient reductions in streams (Hamilton, 2012; Sharply et al., 2013; Van Meter et al., 2016). Furthermore, significant efforts and resources are needed to describe variation in stream nutrient concentrations over time that depict changes in status, whereas biologically based measures integrate data across time and provide evidence for change (Li et al., 2010). Developing tools that increase the probability of detecting changes in highly modified watersheds is important for documenting the effects of management activities and justifying increased or sustained funding.

### Applications with bacterial‐based monitoring

Microorganismal responses to nutrient gradients may provide additional supporting evidence for ecosystem responses to land cover alterations and management efforts (Fasching et al., 2020). Bacterial communities have demonstrated threshold responses to numerous environmental variables, including nutrient enrichment, urbanization, and agricultural intensification (DeVilbiss et al., 2022, 2023; Pilgrim et al., 2022; Simonin et al., 2019). While landscape alterations like urbanization or agricultural intensification can result in habitat simplification and reduced biodiversity of macroinvertebrates through increased deposition of fine sediments (Kaller & Hartman, 2004; Larson et al., 2011), bacterial diversity has been shown to increase with increasing agricultural land use and sedimentation (DeVilbiss et al., 2023). Compared with macroinvertebrates, there is far greater bacterial diversity in stream habitats (7092 bacterial ASVs from 28 sites vs. 334 macroinvertebrate taxa from the calibration dataset, *n* = 99 sites), which increases the likelihood of detecting subtle biological responses to modest improvements in water quality. Further, unlike macroinvertebrates, numerous bacterial species thrive in hypoxic and anoxic conditions typical of highly modified systems like the MAP, as they can leverage alternative terminal electron acceptors to meet energetic needs (Falkowski et al., 2008).

In the MAP, TN‐ and TP‐responsive bacterial ASVs responded consistently and similarly to macroinvertebrate taxa across nutrient gradients, which has been observed in other highly modified regions as well (Simonin et al., 2019). Additionally, the response strength (sum *z*) of nutrient‐intolerant ASVs observed with only 28 samples was similar in magnitude to intolerant macroinvertebrate responses observed with 99 samples, and tolerant bacterial response signals were 3 to 4 times higher than tolerant macroinvertebrate responses. Thus, our results indicate that DNA‐based characterization of bacterial responses to nutrients may provide a more sensitive ecological measure for tracking the nutrient status of streams compared with more traditional assemblages like macroinvertebrates, particularly in highly modified regions where conditions may limit the diversity and responsiveness of macrobiological organisms. Additionally, in situations where sample size may be limited by state monitoring budgets, bacterial monitoring could provide optimal tools for detecting biological responses, particularly as high‐temporal resolution sampling also indicates that microorganismal (diatoms and bacteria) community composition reflects longer term nutrient conditions rather than instantaneous concentrations (Pilgrim et al., 2022; Smucker et al., 2022).

### Conclusion

Assessing stream health in highly modified watersheds poses numerous challenges that can inhibit the detection of robust biological stressor–response relationships. Elevated nutrient gradients and shifted biological tolerance thus require using alternative approaches to more commonly used multimetric indices that consider the full subsidy–stress gradient. By leveraging sensitive taxa‐based analyses like TITAN, we demonstrate that regional biological tolerance databases can be generated and applied to monitor the health and biological recovery of individual streams in response to restoration and management activities in highly modified ecosystems. This approach also works with DNA‐based bacterial datasets and provides consistent and complementary supporting evidence that may be valuable in regions with particularly low macroinvertebrate diversity or where sample sizes are limiting. Our approach is not limited to stream ecosystems and may be useful for monitoring recovery in many ecosystems with limited regional species pools due to widespread perturbation and/or habitat limitation.

## CONFLICT OF INTEREST STATEMENT

The authors declare no conflicts of interest.

## Supporting information


Appendix S1:

Appendix S2:

## Data Availability

Macroinvertebrate community data and water quality data from the calibration dataset that support this research are restricted and not available publicly. Macroinvertebrate community data and water quality data from the calibration dataset are owned by Mississippi Department of Environmental Quality (MDEQ) and are available to qualified researchers by contacting Charles Thompson, Chief, Water Quality Assessments section (cthompson@mdeq.ms.gov) and requesting the Mississippi Benthic Index of Stream Quality (M‐BISQ) macroinvertebrate community and water quality datasets. Macroinvertebrate community data from the validation dataset (DeVilbiss et al., 2024) are available from the USGS ScienceBase‐Catalog: https://doi.org/10.5066/P9X4XXGB. Raw bacterial sequencing data that support this research can be accessed at the NCBI SRA database (https://www.ncbi.nlm.nih.gov/bioproject) under accession number PRJNA909314. Water quality data that support the validation dataset are openly available in the National Water Information System (NWIS) at https://doi.org/10.5066/F7P55KJN (U.S. Geological Survey, 2023). Query information to access the validation water quality dataset is available in Appendix S1.
