# Supplementary material for: From subsidies to stressors: Positively skewed ecological gradients alter biological responses to nutrients in streams
Source: Ecol Appl. 2025 Jan 17;35(1):e3086. doi: 10.1002/eap.3086 (PMC11740167; doi:10.1002/eap.3086)
Supplement: Supplementary file 1 — Appendix S1: [file EAP-35-e3086-s001.pdf]

From subsidies to stressors: Positively skewed ecological gradients alter biological responses to nutrients in streams

Stephen E. DeVilbiss, Jason M. Taylor, Matthew B. Hicks

Ecological Applications

Appendix S1 – NWIS Query Information for validation dataset water quality data

Waterbody Name	Latitude	Longitude	Collection Date
Annis Brake	34.09511	-90.7438	8/2/2021
Ascalmore Creek	33.83889	-90.1322	9/10/2021
Beaver Dam Bayou	33.47886	-90.6189	9/14/2021
Big Sunflower River	34.26815	-90.5653	8/3/2021
Black Bayou	33.98367	-90.5944	8/26/2021
Black Creek	33.70186	-90.0481	8/25/2021
Bobo Bayou	34.31094	-90.1683	9/10/2021
Bogue Phalia	33.44744	-90.8589	9/14/2021
Clear Creek	33.55781	-90.9299	9/14/2021
Dougherty Bayou	33.72424	-90.5801	9/8/2021
Dowling Bayou	32.94371	-90.7278	8/17/2021
Dugan Bayou	33.72339	-90.445	9/9/2021
Edward Bayou	34.01155	-90.7979	8/4/2021
Grancius Bayou	33.15975	-90.9481	8/17/2021
Harris Bayou	34.08533	-90.5818	8/26/2021
Hushpuckena River	33.98714	-90.7115	8/26/2021
Lane Bayou	33.85269	-90.919	9/8/2021
Parks Bayou	33.81814	-90.4233	9/9/2021
Pelucia Creek	33.46611	-90.1325	8/25/2021
Porter Bayou	33.54836	-90.6725	9/14/2021
Potacocowa Creek	33.66889	-90.0728	8/25/2021
Richies Bayou	34.20916	-90.7003	8/2/2021
Sevenmile Bayou	34.59792	-90.2823	8/3/2021
Sherman Creek	34.10403	-90.0787	8/24/2021
Turkey Bayou	33.5266	-90.4137	9/9/2021
White Oak Bayou	34.64459	-90.3354	8/3/2021
