# Supplementary material for: From subsidies to stressors: Positively skewed ecological gradients alter biological responses to nutrients in streams
Source: Ecol Appl. 2025 Jan 17;35(1):e3086. doi: 10.1002/eap.3086 (PMC11740167; doi:10.1002/eap.3086)
Supplement: Supplementary file 2 — Appendix S2: [file EAP-35-e3086-s002.pdf]

From subsidies to stressors: Positively skewed ecological gradients alter biological responses to nutrients in streams

Stephen E. DeVilbiss, Jason M. Taylor, Matthew B. Hicks

Ecological Applications

Appendix S2 – Supplemental Figures

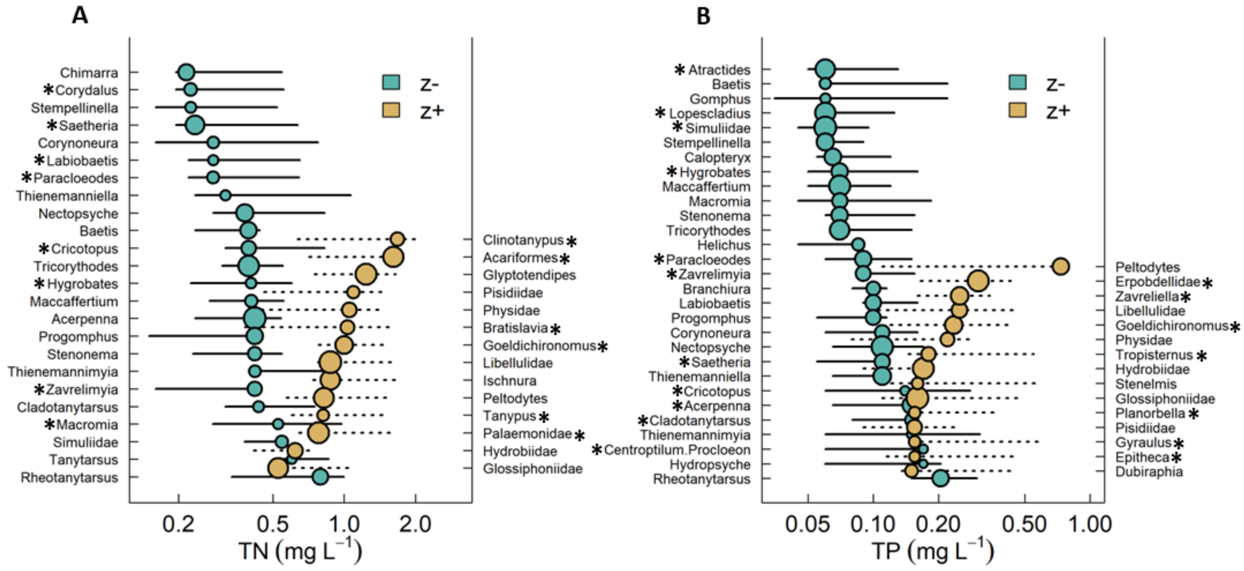

Figure S1. Macroinvertebrate taxa that Threshold Indicator Taxa Analysis (TITAN) identified as tolerant (z+, right axis) and intolerant (z-, left axis) to (A) total nitrogen (TN) and (B) total phosphorus (TP); taxa with an * are found in all ecoregions in the state of Mississippi but only respond to MAP-specific TN and TP gradients. Note, these TITAN results are from analyses in Taylor et al., 2023 (https://doi.org/10.1016/j.ecolind.2023.110377).

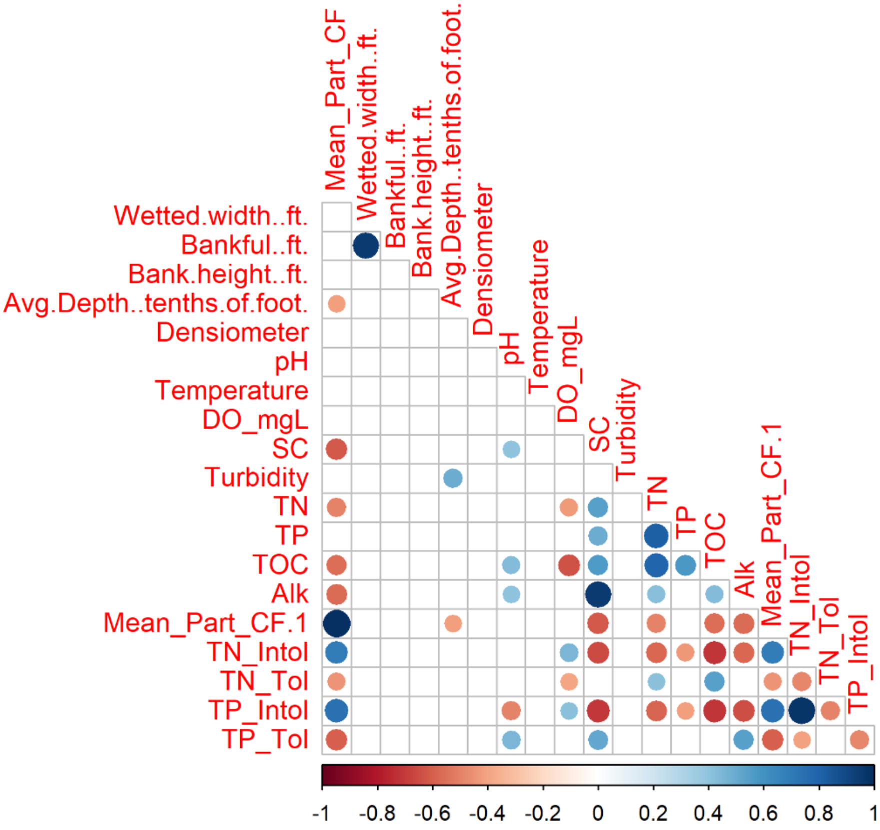

Figure S2. Correlations from the validation dataset of habitat and water quality variables with the richness of nutrient-tolerant and intolerant taxa. TN_Intol = TN-intolerant richness, TN_Tol = TN-tolerant richness, TP_Intol = TP-Intolerant richness, TP_Tol = TP-tolerant richness, Mean_Part_CF = mean particle diameter (µm). Total Nitrogen (TN), Total Phosphorus (TP)

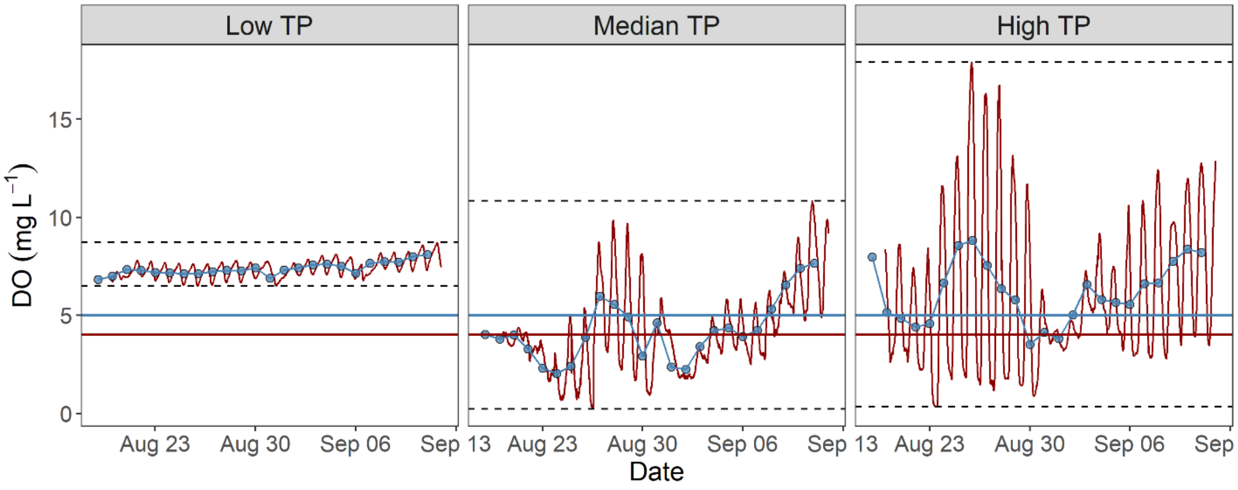

Figure S3. Dissolved oxygen (DO) profiles for 3 streams in the validation dataset that had the minimum (Low TP), median (median TP), and maximum (High TP) total phosphorus (TP) concentrations. The thin red line shows continuous (30 min resolution) DO measurements and the thick, horizontal red line is placed at 4 mg L^{-1} and represents the corresponding Mississippi Department of Environmental Quality's instantaneous DO criteria. The blue circles represent daily averages, and the thick, blue line represents the corresponding 24h average DO criteria of 5 mg L^{-1}. Note how both the range (minimum and maximum shown with dotted black lines) and the number of days that experience DO concentrations (as shown with the continuous data) below the state criteria increase as TP concentration increases.

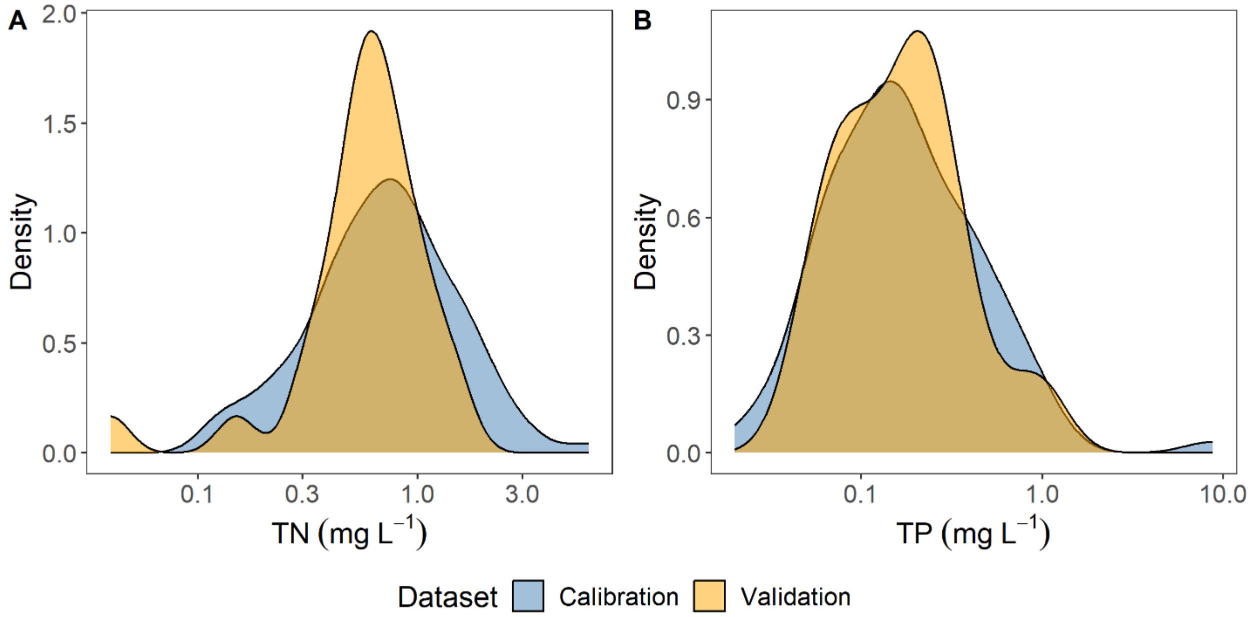

Figure S4. Distribution of total nitrogen (TN, panel A), and total phosphorus (TP, panel B) observations in the calibration (blue) and validation (orange) datasets.
